# The tumor microenvironment of colorectal cancer: stromal TLR-4 expression as a potential prognostic marker

**DOI:** 10.1186/1479-5876-8-112

**Published:** 2010-11-08

**Authors:** Rosaria Cammarota, Valentina Bertolini, Giuseppina Pennesi, Eraldo O Bucci, Ornella Gottardi, Cecilia Garlanda, Luigi Laghi, Massimo C Barberis, Fausto Sessa, Douglas M Noonan, Adriana Albini

**Affiliations:** 1 Oncology Research Laboratory, Science and Technology Park, IRCCS MultiMedica, (via Fantoli 16/15), Milan, (20138), Italy; 2 Department of Pathology, Science and Technology Park, IRCCS MultiMedica, (via Fantoli 16/15), Milan, (20138), Italy; 3 Department of Oncology, IRCCS MultiMedica, (via Piemonte 70), Castellanza, (21053), Italy; 4 Istituto Clinico Humanitas IRCCS, (via Manzoni 56), Rozzano, (20089), Italy; 5 Department of Pathology, Istituto Europeo Oncologico, (via Ripamonti 435), Milan, (20141), Italy; 6 Department of Experimental Medicine, Università degli Studi dell'Insubria, (viale Ottorino Rossi n9), Varese, (21100) Italy

## Abstract

**Background:**

Colorectal cancer can be efficiently treated when found at early stages, thus the search for novel markers is of paramount importance. Since inflammation is associated with cancer progression and angiogenesis, we investigated expression of cytokines like IL-6 and other mediators that play a key role in the innate immune system, in particular toll like receptor 4 (TLR4), in the microenvironment of lesions from different stages of colon disease progression, from ulcerative colitis to adenoma and adenocarcinoma to find useful markers.

**Methods:**

The presence of inflammatory cells and expression of key cytokines involved in the inflammation process were quantified by immunohistochemistry in specific tissue compartments (epithelial, stromal, endothelial) by immunohistochemistry. A murine azoxymethane/dextran sulfate model in which Tir8, a negative regulator of the inflammatory response, was ablated was used to confirm the clinical observations. 116 Archival tissue samples from patients with different stages of colorectal disease: 13 cases of ulcerative colitis (UC), 34 tubular or tubulo-villous adenomas (AD), and 53 infiltrating adenocarcinomas. 16 specimens of healthy mucosa surgically removed with the cancerous tissue were used as a control.

**Results:**

The differences between healthy tissues and the diverse lesions was characterized by a marked inflammatory-angiogenic reaction, with significantly (P < 0.05) higher numbers of CD68, CD15, and CD31 expressing cells in all diseased tissues that correlated with increasing grade of malignancy. We noted down-regulation of a potential modulator molecule, Hepatocyte Growth Factor, in all diseased tissues (P < 0.05). TLR-4 and IL6 expression in the tumor microenvironment were associated with adenocarcinoma in human samples and in the murine model. We found that adenocarcinoma patients (pT1-4) with higher TLR-4 expression in stromal compartment had a significantly increased risk in disease progression. In those patients with a diagnosis of pT3 (33 cases) colon cancer, those with very high levels of TLR-4 in the tumor stroma relapsed significantly earlier than those with lower expression levels.

**Conclusions:**

These data suggest that high TLR-4 expression in the tumor microenvironment represents a possible marker of disease progression in colon cancer.

## Background

Colorectal carcinoma (CRC) is the fourth most frequent cause for death from cancer worldwide. Disparate factors increase a person's risk of developing the tumor, such as age, inflammatory bowel disease, personal and/or family (such as hereditary nonpolyposis colorectal cancer; HNPCC) history of colorectal tumors (adenoma or adenocarcinoma), and environmental factors [1-3]. The molecular genetic alterations along the process leading to colon cancer is one of the best characterized of all the processes in cancer progression [4]. However, much less is known concerning the role of the tumor microenvironment of CRC [5]. The development of a tumor alters the homeostasis of the surroundings tissues engaging diverse mechanisms; key among these is the activation of inflammation and of innate and adaptive arms of the immune response [6,7]. The observations that many tumors contain numerous inflammatory leukocytes, and that chronic inflammation predisposes to certain cancers, particularly colorectal cancer, historically led to develop the concept of a functional link between chronic inflammation and cancer [8].

Chronic inflammation could promote colon carcinogenesis by inducing gene mutations, inhibiting apoptosis or stimulating angiogenesis and cell proliferation [9], as well as inducing epigenetic alterations associated with cancer development. In spite of this extensive evidence indicating a role for inflammation in both colon cancer insurgence and progression, there is relatively little information on inflammation-associated microenvironmental changes associated with hyperplasia/neoplasia development and its evolution towards invasive colorectal adenocarcinoma. Tumors produce molecules that attract a constant influx of inflammatory cells. Recent studies have shown that immune cell infiltration of dysplastic lesions, based on pan-leukocyte CD45 staining, increases with increasing malignancy of the lesions, including breast, prostate and skin cancer development [10-12]. Once within the tumor microenvironment, these cells are polarized toward an alternative activation [8] where they can stimulate initiated cell proliferation, stromal disruption, and tumor growth [13,14]. Currently, there is increasing evidence that the innate immune system plays a key role in orchestrating angiogenesis in cancer, producing angiogenic factors that enhance endothelial cell recruitment, proliferation and new vessel formation [15-18], contributing to tumor promotion and other pathological conditions [12,13,15-17,19]. Although chronic inflammatory conditions clearly predispose to CRC, and use of anti-inflammatory agents can prevent adenomas [20,21] and CRC [22,23], the role of immune cell infiltration into CRC is controversial, as some studies have suggested that increased immune cell infiltration is beneficial [24,25].

Several cytokines appear to correlate with CRC progression, key among these is IL-6, an inflammatory cytokine secreted in response to damage. IL-6 levels are increased in most epithelial tumors [26], and high serum IL-6 levels have been found to correlate with a poor clinical prognosis in patients with diverse carcinomas (renal, ovarian and colorectal) [27-30]. Given the observed involvement of IL-6 and its downstream targets in the regulation of cell proliferation, survival, and metabolism, it is not surprising that IL-6 signaling has also been implicated in tumorigenesis [31], and it has been suggested that it has a possible oncogenic role, driving expression of central hubs in cancer such as STAT3 [32]. IL-6 is a downstream product of activation of NF-κB, a fundamental molecular hub linking inflammation and cancer [33]. IL-6 is a key mediator in a mouse model of microbially induced CRC [34]. NF-κB and IL-6 expression is induced by activation of specific pattern recognition receptors, such as Toll-Like Receptor 4 (TLR-4) [35]. TLR-4 is a transmembrane pattern recognition receptor that provides a critical link between immune stimulants produced by microorganisms, in particular lipopolysaccharide, and the initiation of the innate immune reaction to foreign agents, but also to tumor cells [36]. TLR-4 has been found to be expressed by leukocytes [37], endothelial cells [38], and epithelial cells [39]. In the gut, activation of TLR-4 in enterocytes leads to an inhibition of enterocyte migration and proliferation as well as to the induction of enterocyte apoptosis-factors that would be expected to promote intestinal injury while inhibiting intestinal repair. Moreover, epithelial TLR signaling, acting in concert with TLR signaling by leukocytes, participates in the development of intestinal inflammation [40]. Activation of TLR-4 leads to induction of an inflammatory response mediated by multiple pathways and stimulates the production of numerous cytokines, in particular IL-6 [35]. It has been also demonstrated that TLR-4 signaling is crucial for colon carcinogenesis in chronic colitis, being responsible for induction of COX-2, increased prostaglandin E2 production, and activation of EGFR phosphorylation in chronic colitis [21,41-43]. Since in previous studies we reported that TLR-4 levels were up-regulated in the thymus of myasthenia gravis patients [44], suggesting an innate-immune mediated priming for subsequent autosensitization to the acetylcholine receptor, we investigated the expression of IL-6 and TLR-4 across a spectrum of tissues recapitulating diverse steps along the evolution towards colon cancer. The investigated tissues included resection margins from radical surgery (R0, presumed to be healthy, although field effects cannot be ruled out), inflamed mucosa from patients with ulcerative colitis, adenomas and adenocarcinomas. We examined 3 specific compartments in each tissue, the epithelial compartment, the stroma and endothelial compartment. Additionally, we studied tumor tissues derived from animals lacking Tir8, an interleukin-1/Toll-like receptor family member highly expressed in the intestinal mucosa [45] in the azoxymethane and dextran sulfate sodium salt (DSS) model of CRC. In this mouse model of colonic carcinigenesis, the lack of constraints to NF-κB driven inflammation, mediated via interleukin-1 inhibition, allows investigation of the effects of enhanced inflammation.

We observed a strong correlation between the increased expression of IL-6 and TLR-4 with increasing tissue dysplasia up to malignancy, higher TLR-4 and IL-6 was also found in tumor tissues derived from animals lacking Tir8 as compared to wild-type controls. Hepatocyte Growth Factor (HGF) was markedly down-regulated in all the diseased tissues (ulcerative colitis, adenoma or adenocarcinoma) studied.

As these data suggested involvement of innate immune mediated mechanisms, we also examined markers representative of the innate immune network involved in tumor reactive inflammation and inflammation-driven angiogenesis, including: CD31, expressed on continuous endothelia and is a surface receptor for activated leukocytes that favors leukocyte diapedesis [46]; CD68, highly expressed in monocytes and tissue macrophages and involved in endocytosis and lysosomal trafficking [47]; and CD15, also known as Lewis X, a marker for mature granulocytes suggested to increase the growth of tumor cells [48]. We observed a strong correlation between the increased expression of these inflammation markers and increasing tissue dysplasia up to malignancy.

## Materials And Methods

### Patient samples

This study was conducted on 116 formalin fixed and paraffin embedded tissue blocks corresponding to samples from four different steps of disease progression: 13 cases of ulcerative colitis (UC), 34 tubular or tubulo-villous adenomas with low (29 cases) to high (5 cases) grade dysplasia (AD), and 53 infiltrating adenocarcinomas classified using TNM (ajcc, american joint committee on cancer, VI edition) with T1 (7 cases,), T2 (10 cases), T3 (33 cases), and T4 (3 cases) (AC) (complete patient characteristics are in Additional file 1, Supplemental Table S1). Sixteen specimens of healthy mucosa (R0, radical resection margins) surgically removed with the cancerous tissue were used as a control. For the adenocarcinoma patients, follow-up of up to 9 years was available.

### Animal model of colitis-associated cancer in Tir8-/- mice

Tir8-deficient (Tir8-/-) mice were generated as previously described [49]. We used 8-12 week old mice on a C57Bl/6 (H2^b^) genetic background. C57Bl/6 (Tir8+/+) were used as wild-type (WT) controls. To induce colon tumors, mice were treated with azoxymethane followed by three cycles of 1.5% DSS as previously described [24].

Briefly, a single dose (10 mg/kg) of the mutagenic agent azoxymethane (Sigma) was injected in Tir8-/- and wild type (WT) control mice, followed by 3 cycles of 3%, 2%, or 1.5% DSS (molecular mass, 40 kDa; ICN) dissolved in sterile, distilled drinking water. At the end of the treatment, after 60 days, mice were euthanized, the large intestine was removed, open longitudinally, rinsed and "rolled" and processed for histological and immunohistochemistry analysis, providing a complete spectrum of the length of the large intestine in each section. Research projects involving animals were first approved by Italian National Institute of Health (ISS), then experiments were performed following protocol registered with number 18/17/2004, approved by Istituto Clinico Humanitas (ICH) ethical committee. The care and use of the animals were in compliance with laws of the Italian Ministry of Health (D.L. N. 116/1992) and the guidelines of the European Community.

### Histological analysis and immunohistochemistry

Three micrometer tissues of the paraffin-embedded sections of human specimens were mounted on slides coated with silane (Dako, Milan, Italy) and stained with hematoxylin for histological analysis. For analysis of murine tissues, after sacrifice the large intestines of the treated mice were removed, fixed in 10% neutral buffered formalin, and embedded in paraffin. Three-micrometer-thick consecutive sections that covered the entire length of the "rolled" colon were cut and mounted on silanized slides.

Hematoxylin-Eosin staining (H&E) was performed according to standard protocols. For immunohistochemistry, slides were deparaffinized in xylene and rehydrated in a series of graded alcohols, and the antigen was retrieved in 0.01 mol/L sodium citrate buffer or EDTA ph 8 0.5M. Sections were then treated with 3% of hydrogen peroxide to inhibit endogenous peroxidase. The sections were stained with primary antibodies, listed in Table 1, followed by appropriate secondary antibody, then the Dako REAL EnVision system, Peroxidase/DAB+, Rabbit/Mouse was used as revelation system according to the manufacturer's recommendations. The reaction was visualized by use of the appropriate substrate/chromogen (Diaminobenzidine, DAB) reagent. Counterstaining was performed using Mayer's hematoxylin (Sigma, Taufkirchen, Germany).

**Table 1:** Primary antibodies used for immunohistochemical detection.

Primary antibody	Species raised in	Species directed to	Supplier (clone and/or #)	Dilution
CD68	Mouse	Human	DAKO (PG-M1, #M0876)	1: 100

CD15	Mouse	Human	DAKO (C3D-1, #M0733)	1: 20

CD31	Mouse	Human	DAKO (JC70A, #M0823)	1: 20

TLR-4	Rabbit	Human	SANTA CRUZ BIOTECHNOLOGY (M-300, #sc-30002) (Santa Cruz, California, USA)	1: 100
		
		Mouse	NOVUS BIOLOGICALS (NB100-56581) (Littleton, CO, USA)	1:600

HGF	Goat	Human	SIGMA (H7157)	1: 400

IL-6	Rabbit	Human	NOVUS BIOLOGICALS (NB600-1131)	1: 400
		
		Mouse	ABCAM (ab6672) (Cambridge, UK)	1:400

### Image acquisition and rendering

Bright field images of H&E and antibody-stained sections were visualized with a Nikon E800light microscope, and photomicrographs taken at a 400× magnification using a digital image acquisition system.

### Quantification of staining and statistical analysis

Positive staining was identified when the epithelial, endothelial cells or stroma showed clear brown staining and quantified by counting the positive cells in 3 representative areas for each section, and expressing this as a percentage of average number of positive cells/section. Stroma was defined as the connective tissue areas around the tumor cells along with any immune infiltrate in these areas that were clearly not epithelial or tumor cells, or vessels. Statistical differences between individual cell groups were determined using an unpaired two way t-test (Mann-Whitney) where P values < 0.05 were considered statistically significant. Regression analysis was also performed to test statistical significance of correlation between the expression of selected markers. P values < 0.05 were considered statistically significant. All data were analyzed using the Prism (Graph Pad) statistics and graphing program.

Disease free survival (time from diagnosis to relapse, progression or death of disease) were estimated for each marker by means of Kaplan Meier method for patients with CRC using the Survival Analysis System Excel addin by SG Shering, Univ College of Dublin. The median value of the percentage of expression for each marker in any tumor compartment was used as cut-off. Statistical differences between groups were evaluated by the Log-rank test.

## Results

### Expression of cytokines and TLR-4 in specific tissue compartments

**Figure 1:**
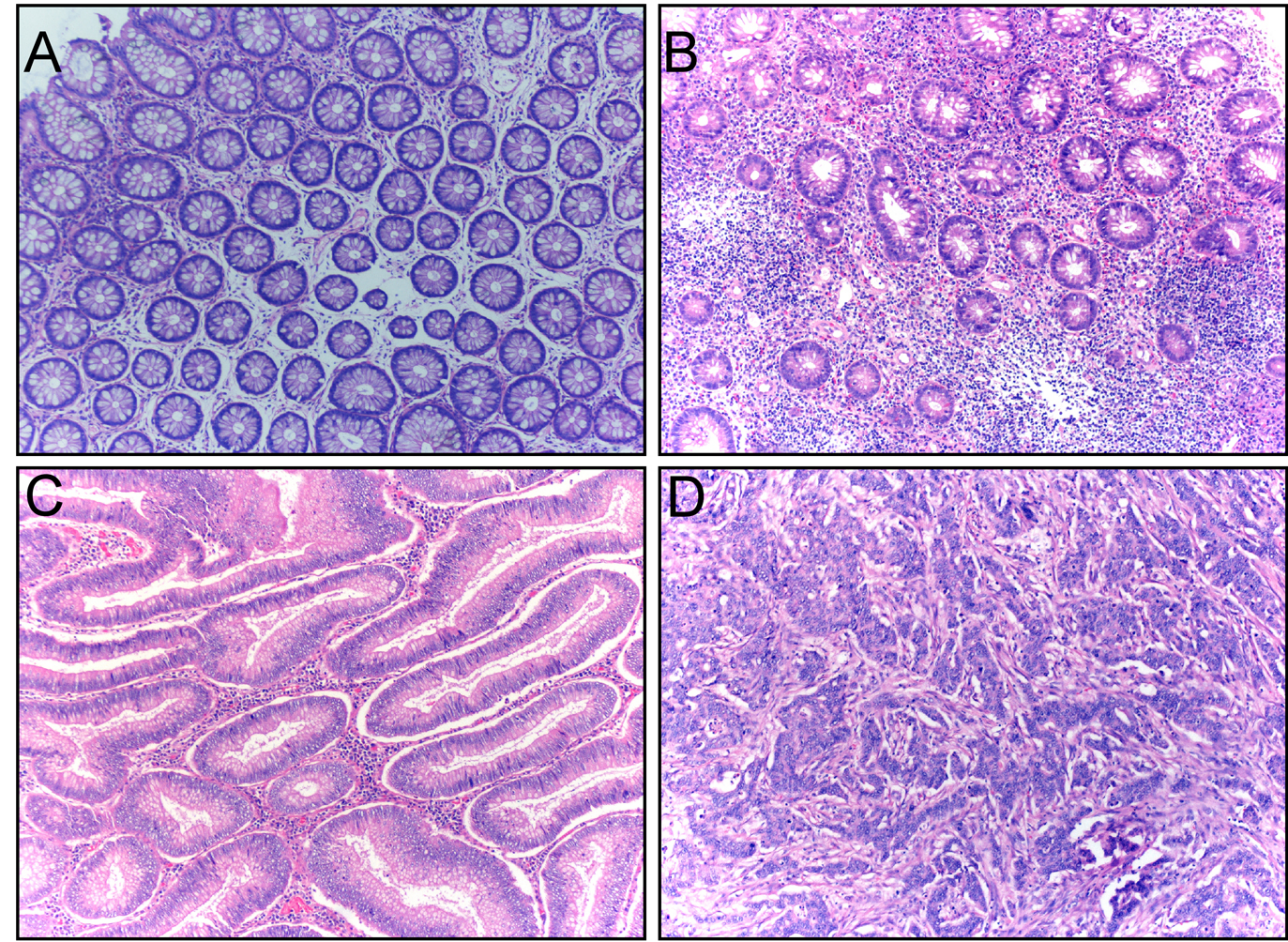
**Hematoxylin and Eosin staining**. Examples of hematoxylin and eosin (H&E) staining of healthy tissues (A), ulcerative colitis (B), adenomas (C) and adenocarcinomas (D) (magnification ×100).

The histological features of different types of disease analyzed in this study are shown in figure 1, where the different degrees of malignancy are apparent. We could readily discern 3 specific compartments within each tissue, an epithelial compartment, an endothelial compartment (confirmed by CD31 staining, see below) and a stromal compartment, and immuno-reactivity was examined within each compartment (Table 2).

**Table 2:** Percentage of cells present within each tissue compartment.

Condition	Healthy % (+ SEM)			Ulcerative colitis (UC) % (+ SEM)			Adenoma (AD) % (+ SEM)			Adenocarcinoma (AC) % (+ SEM)		
**Compartment**	**Endothelium**	**Epithelium**	**Stromal**	**Endothelium**	**Epithelium**	**Stromal**	**Endothelium**	**Epithelium**	**Stromal**	**Endothelium**	**Epithelium**	**Stromal**

**Marker**												

**CD31**	6.7 (± 0.5)	n.d.	n.d.	10.5(± 0.5)	n.d.	n.d.	11.2(± 0.9)	n.d.	n.d.	14.6(± 1)	n.d.	n.d.

**HGF**	n.d.	35.1(± 2.1)	n.d.	n.d.	15.5(± 1.3)	n.d.	n.d.	19.3(± 1.5)	n.d.	n.d.	17.3(± 1.5)	n.d.

**CD68**	n.d.	n.d.	8.7(± 1.0)	n.d.	n.d.	18(± 0.8)	n.d.	n.d.	23(± 2.3)	n.d.	n.d.	26.6 (± 1.8)

**CD15**	1.4(± 0.3)	1 (± 0.4)	2.5(± 0.6)	2.6(± 0.2)	4.7(± 0.7)	7.0(± 1.3)	3.3(± 0.4)	11.6(± 2.4)	16.(± 1.9)	3.6(± 0.6)	13.4(± 1.0)	22.8(± 2.5)

**TLR-4**	2.4(± 0.3)	3.6(± 0.9)	5.8(± 1.5)	4.0(± 0.4)	8.2(± 1.6)	16.1(± 1.5)	6.6(± 0.5)	16.8(± 2.5)	25.4(± 2.8)	8.0(± 0.8)	19.6(± 3.3)	28.2(± 2.7)

**IL-6**	2.9(± 0.2)	4.9(± 0.5)	7.06(± 0.2)	4.1 (± 0.3)	11.2(± 1.2)	17.2(± 1.4)	6(± 0.4)	21.5(± 2.0)	27.9(± 3.2)	6.8(± 0.8)	32.0(± 3.0)	34.6(± 2.7)

Shown are the averaged percentages of cells (± Standard Error of the Mean-SEM) positive for staining within each tissue compartment. UC = ulcerative colitis; AD = adenomas; AC = adenocarcinomas; n.d. = not detected.

**Figure 2:**
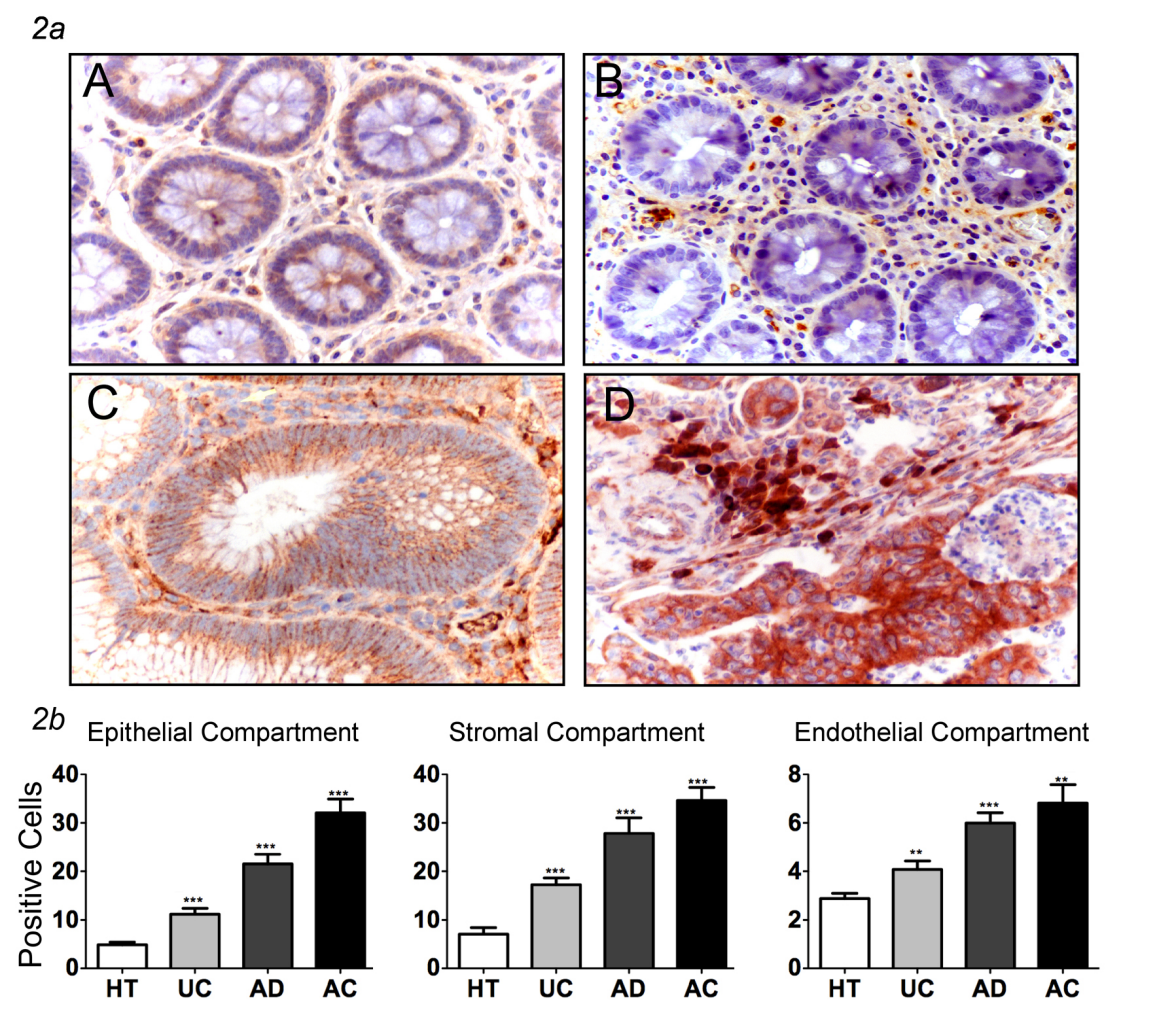
**IL-6 expression in human colon tissues**. ***2a ***Expression of IL-6 in normal healthy tissues (A), ulcerative colitis (B), adenomas (C) and adenocarcinomas (D); some scattered epithelial and stromal cells are stained with weak intensity. In the dysplastic conditions there is an increased staining (magnification ×400). ***2b ***Different expression of IL-6 in endothelial, epithelial and stromal compartments show that in all groups this marker is significantly increased respect to healthy tissues (mean ± SEM; **P < 0.01, *** P < 0.001). HT = healthy tissues (N = 16); UC = ulcerative colitis (N = 13), AD = adenomas (N = 34; 29 low and 5 high grade), AC = adenocarcinomas (N = 53; 7 T1, 10 T2, 33 T3, 3 T4).

The immuno-reactivity for IL-6 was mostly observed in the epithelial and stromal compartments (Figure 2, Table 2). The frequency of IL-6-producing cells within the epithelial and stromal compartments of healthy colon tissues were 4.9% and 7.1%, respectively. The initiation of the neoplastic process corresponded to an expansion of IL-6+ cells in these tissue compartments, rising to 11.2% and 17.3% in UC specimens, to 21.5% and 27.9% in AD, and, finally, to 32% and 34.6% in AC. The observed values in the diseased tissues were statistically different when compared with the values of healthy specimens (p < 0.05) (Figure 2, Table 2). This trend was also confirmed in the endothelial compartment of healthy and tumor tissues, with the comparison between IL-6+ cells in AC (6.8%) and healthy tissue (2.9%) being significant (p < 0.05) (Figure 2, Table 2).

**Figure 3:**
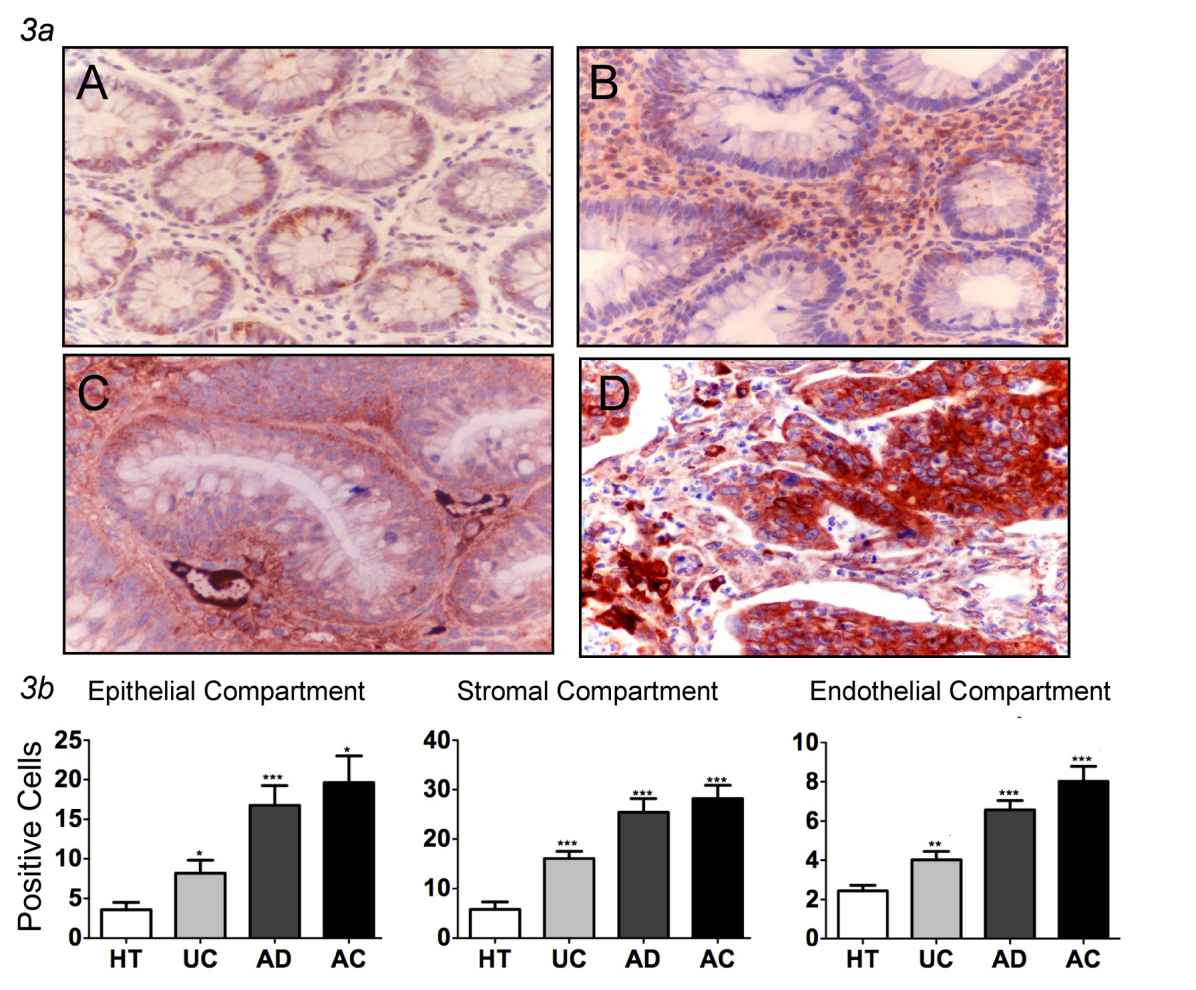
**Expression of TLR-4 in human colon tissues**. ***3a ***TLR-4 immunohistochemistry analysis. Different expression of TLR-4 in healthy tissues (A), ulcerative colitis (B), adenomas (C) and adenocarcinomas (D) show that increasing grade of dysplasia directly correlates with higher expression of this marker (magnification ×400). ***3b ***Different expression of TLR-4 in endothelial, epithelial area and stromal department shows that in all groups TLR-4 is significantly increased respect to healthy tissues (mean ± SEM; **P < 0.01, *** P < 0.001). HT = healthy tissues (N = 16); UC = ulcerative colitis (N = 13), AD = adenomas (N = 34; 29 low and 5 high grade), AC = adenocarcinomas (N = 53; 7 T1, 10 T2, 33 T3, 3 T4).

TLR-4+ cells were preferentially distributed within the epithelial and stromal compartments of all the specimens, although in different percentages (Figure 3, Table 2). The increased presence of TLR-4-expressing cells corresponded to an increasing grade of dysplasia (Figure 3). In particular, the averaged percentage of TLR-4+ cells in the epithelial and stromal compartments of healthy tissues were 3.6% and 5.8%, respectively. These values increased to 8.2% and 16.1% within the epithelial and stromal areas of UC specimens (p < 0.05). In AD tissues the TLR-4+ cells rose to 16.8% and 25.4%, respectively (p < 0.05 when compared with percentage of TLR-4+ cells in healthy tissues and UC), while in AC their levels additionally increased up to five times (19.6% and 28.2%, in the epithelial and stromal compartments, respectively; p < 0.05). In the stroma, TLR-4 expression was largely due to immune cells showing a morphology typical of macrophages [50,51].

When we examined the endothelial compartment, we also found a trend towards an increase in TLR-4-expressing cells paralleling tumor progression. In particular, the percentage of positive cells in AD and AC (6.6% and 8.0%, respectively) significantly differed from the values observed in the endothelium of healthy tissues (2.44%) (p < 0.05 for all comparisons) (Figure 3, Table 2).

In pT3 AC (33 cases), a positive correlation was observed between the expression of IL-6 and the presence of TLR-4+ cells in the stromal and epithelial compartment (R^2 ^= 0.16, p < 0.05, and R^2 ^= 0.33, p < 0.05, respectively), and between the expression of IL-6 and the presence of CD15+ cells in the stromal compartment (R^2 ^= 0.23, p < 0.05).

**Figure 4:**
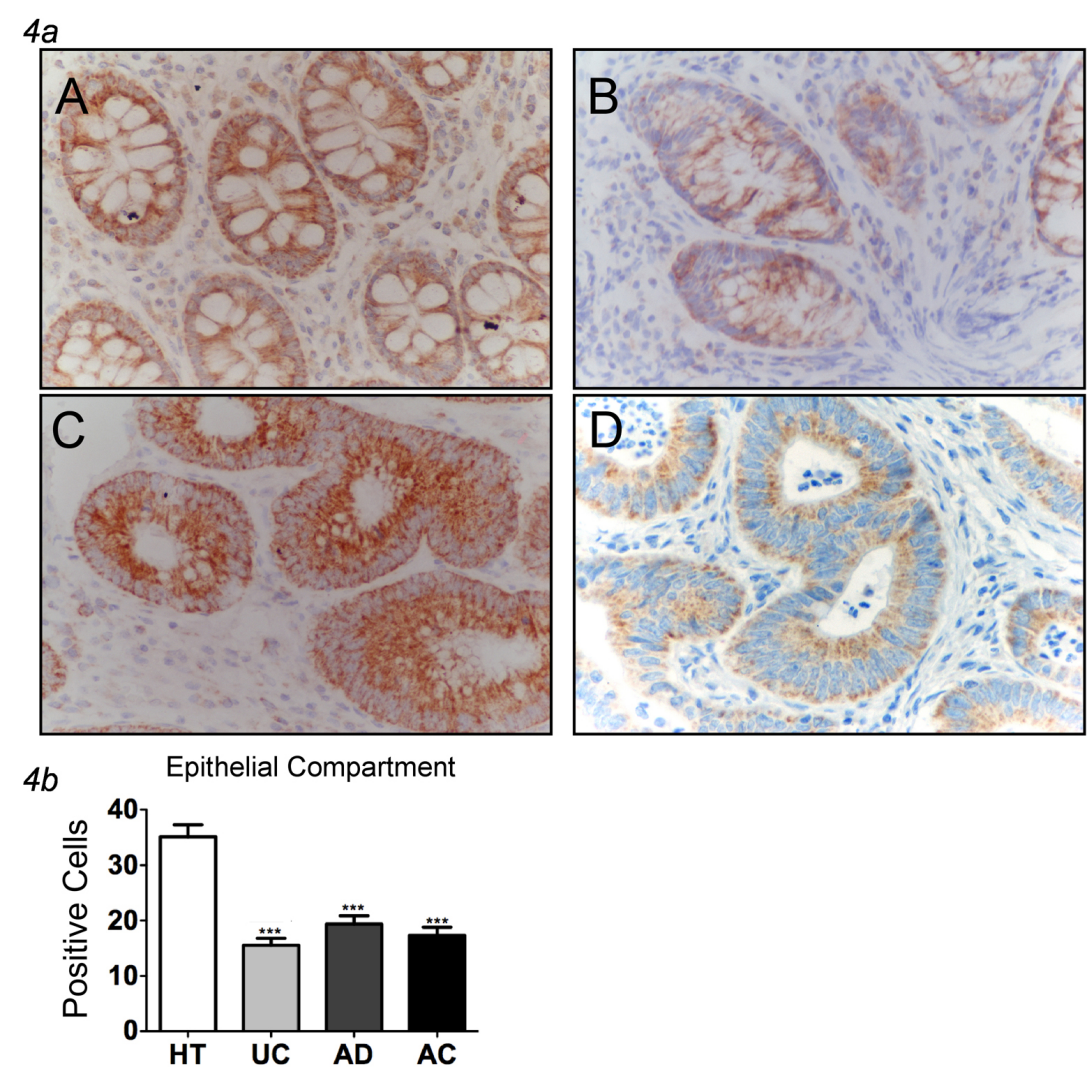
**Expression of HGF in human colon tissues**. ***4a ***Different expression of HGF (present only in epithelial compartment) in healthy tissues (A), ulcerative colitis (B), adenomas (C) and adenocarcinomas (D). The peak of immunoreactivity is in the healthy tissue. In contrast, in the dysplastic lesions, there is a drop in expression as the grade of dysplasia increases. The lowest expression is in UC cases (magnification ×400). ***4b ***Expression of HGF in healthy tissues (HT), UC, AD and AC. In all groups HGF is significantly reduced respect to healthy tissues (mean ± SEM; **P < 0.01, *** P < 0.001). HT = healthy tissues; UC = ulcerative colitis, AD = adenomas, AC = adenocarcinomas.

A very different, inverse trend was observed analyzing the frequency of HGF-secreting cells in healthy and pathological specimens. We observed the highest number of HGF+ cells in healthy tissues (35.1%), the incidence of HGF+ cells decreased sharply in specimens of UC (15.5%, p < 0.05), AD (19.4%, p < 0.05), and AC (17.3%, p < 0.05) (Figure 4, Table 2). Notably, in tumor sections, the HGF positive cells were limited to the epithelial compartment, suggesting that HGF in the colon is a marker of an intact normal epithelium, and is down-regulated during the inflammatory response.

### Expression of IL-6 and TLR-4 in a murine CRC model

**Figure 5:**
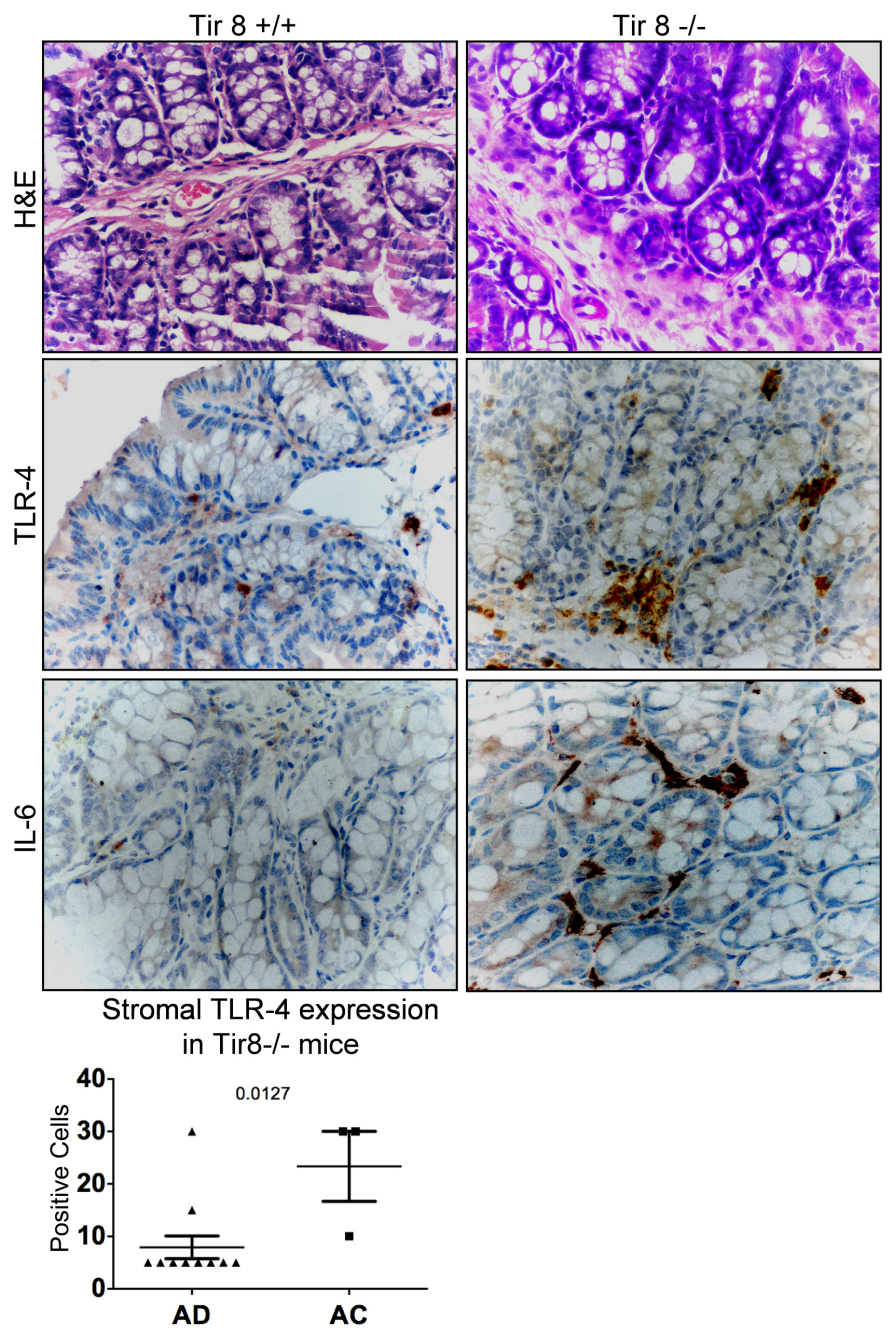
**Staining in murine models of colorectal cancer**. Comparison of H&E, TLR-4 and IL-6 immunostaining in mice wild type and knock-out for Tir8. Tir8 -/- mice had a higher grade of dysplasia and an increased expression of TLR-4 and IL-6 than wt mice (magnification ×400).

We compared our findings on IL-6 and TLR-4 in human tissues with inflammation-tracking in a mouse model of experimentally-induced colitis-associated cancer in Tir8 deficient mice. After treatment with the azoxymethane and DSS carcinogenic regimen, all mice developed tumors, regardless of genetic background. However, a higher numbers of lesions developed in the Tir8 -/- mice, and these lesions were higher grade adenomas as compared to those that developed in the WT mice, consistent with previous reports [45]. Immuno-histochemical analyses of the colons from WT and Tir8 -/- mice indicated higher staining for TLR-4 and IL-6 in the neoplastic tissues of specimens from Tir8-deficient mice as compared with neoplastic tissues from WT animals (Figure 5a). Moreover, in KO mice, the values of TLR-4 in the stromal compartment (again associated with cells of macrophage morphology) of the adenocarcinomas were elevated and statistically different form the values of adenoma specimens (p < 0.05) (Figure 5b).

Similar results were observed when we compared the values of the adenomas of both WT and KO mice with the adenocarcinomas that developed only in KO mice (data not shown).

### Relationship between TLR-4 and disease-free survival time

**Figure 6:**
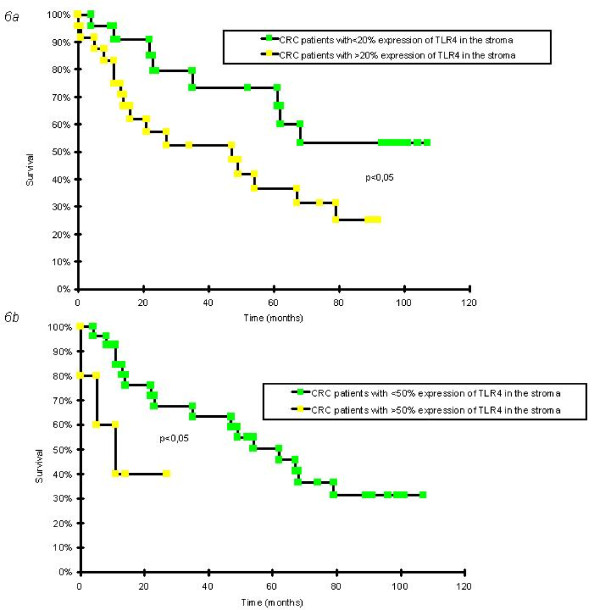
**Disease Free Survival (DFS) curves of adenocarcinoma patients associated with TLR-4 expression in the tumor stroma**. Disease free survival (time from diagnosis to relapse, progression or death of disease) were estimated for each marker by means of Kaplan Meier method for patients with CRC using the Survival Analysis System Excel addin by SG Shering, Univ College of Dublin. **6a **DFS curve of all adenocarcinoma patients (pT1-4) (53 cases). CRC patients with a low percentage of TLR-4+ cells in the tumor stromal compartment (less than the median value corresponding to 20% of the cells positive) relapsed with a greater time interval and several showed survival of over 100 months, while those patients with a percentage of TLR-4+ cells in the stromal compartment higher than the median value relapsed earlier and fewer showed long term survival (RR 2.36; log rank chi-square 4.25, p < 0.05). **6b **DFS curve of patients with adenocarcinoma at the pT3 (33 cases) stage. Patients with a percentage of TLR-4+ cells in the tumor stromal compartment more than 50% relapsed early (within 14 months), while those with a percentage of TLR-4+ cells expression less than 50% relapsed much later (within 40 months, RR 3.15; log rank chi-square 4.03, p < 0.05).

Given the consistent relationship between expression and progression of IL-6 and TLR-4 in human samples and murine models, we evaluated the disease free survival time of patients affected by CRC as a function of marker expression in each tissue compartment. Statistically significant results were obtained for TLR-4 expression in the tumor stroma compartment. In particular, we observed that CRC patients (adenocarcinomas, pT1-4) with a percentage of TLR-4+ cells in the tumor stromal compartment lower than the median value (20% of the cells positive) relapsed with a greater time interval and several showed survival of over 100 months, while those patients with a percentage of TLR-4+ cells in the stromal compartment higher than the median value relapsed earlier and fewer showed long term survival (RR 2.36; log rank chi-square 4.25, p < 0.05) (Figure 6a).

We then examined the largest group, adenocarcinoma pT3 patients (33 cases), using as cut off a high percentage of expression (≥50% of the cells positive) we discriminated two different trends. Again, those patients with the highest TLR-4 expression relapsed early (within 14 months), while those with lower expression relapsed much later (within 40 months, RR 3.15; log rank chi-square 4.03, p < 0.05) (figure 6b).

Given the relationship between TLR-4 expression and survival in adenocarcinomas, and the general tendency towards increased inflammatory markers as a function increasing tissue dysplasia up to malignancy, we then investigated several markers of inflammatory cells and angiogenesis.

### Expression of inflammation markers with increasing tissue dysplasia

**Figure 7:**
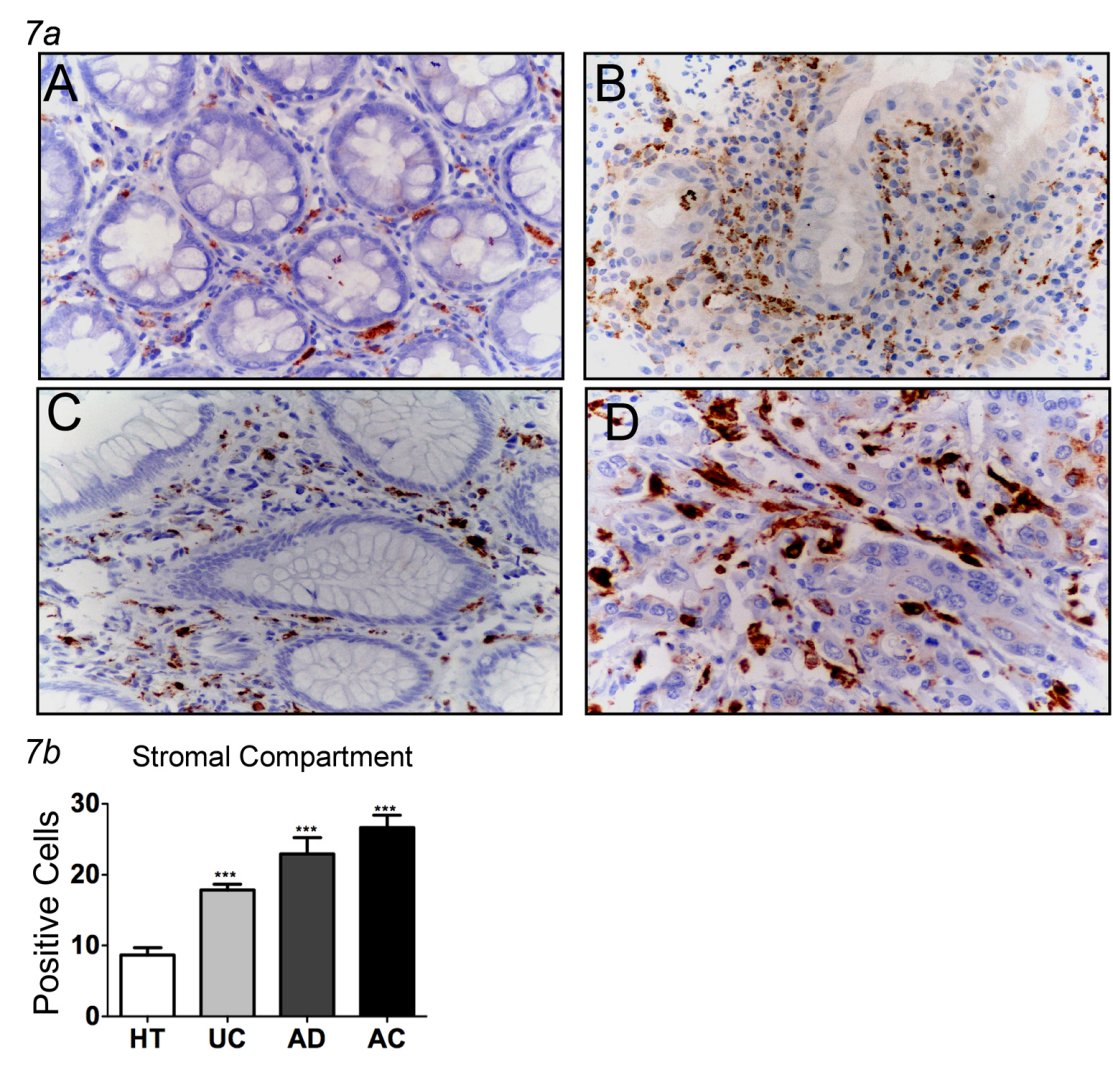
**CD68 immunostaining in human colon tissues**. ***7a ***CD68 immunostaining in healthy tissues (A), ulcerative colitis (B), adenomas (C) and adenocarcinomas (D) shows a growing expression of intensity, percentage of positive cells and density in stromal compartment (magnification ×400). ***7b ***Expression of CD68 in the stromal compartment of the different groups. In all groups CD68 is significantly increased respect to healthy tissues (mean ± SEM; **P < 0.01, *** P < 0.001). HT = healthy tissues (N = 16); UC = ulcerative colitis (N = 13), AD = adenomas (N = 34; 29 low and 5 high grade), AC = adenocarcinomas (N = 53; 7 T1, 10 T2, 33 T3, 3 T4).

Immunohistochemical analysis showed that CD68+ cells progressively colonized the tumor stroma, being almost absent in the healthy tissue, clearly present in pre-cancerous conditions, and peaking in samples from patients with adenocarcinomas (Figure 7, Table 2). In particular, positive staining for CD68 was 8.7% in healthy tissue, 17.9% in samples from patients with UC, 23.0% in AD, and 26.6% in AC (all p < 0.05, as compared to healthy tissue). A statistically significant difference was also observed comparing the percentage of CD68+ cells between specimens of UC and AC (p < 0.05). The staining pattern was consistent with localization to macrophages within the stroma.

**Figure 8:**
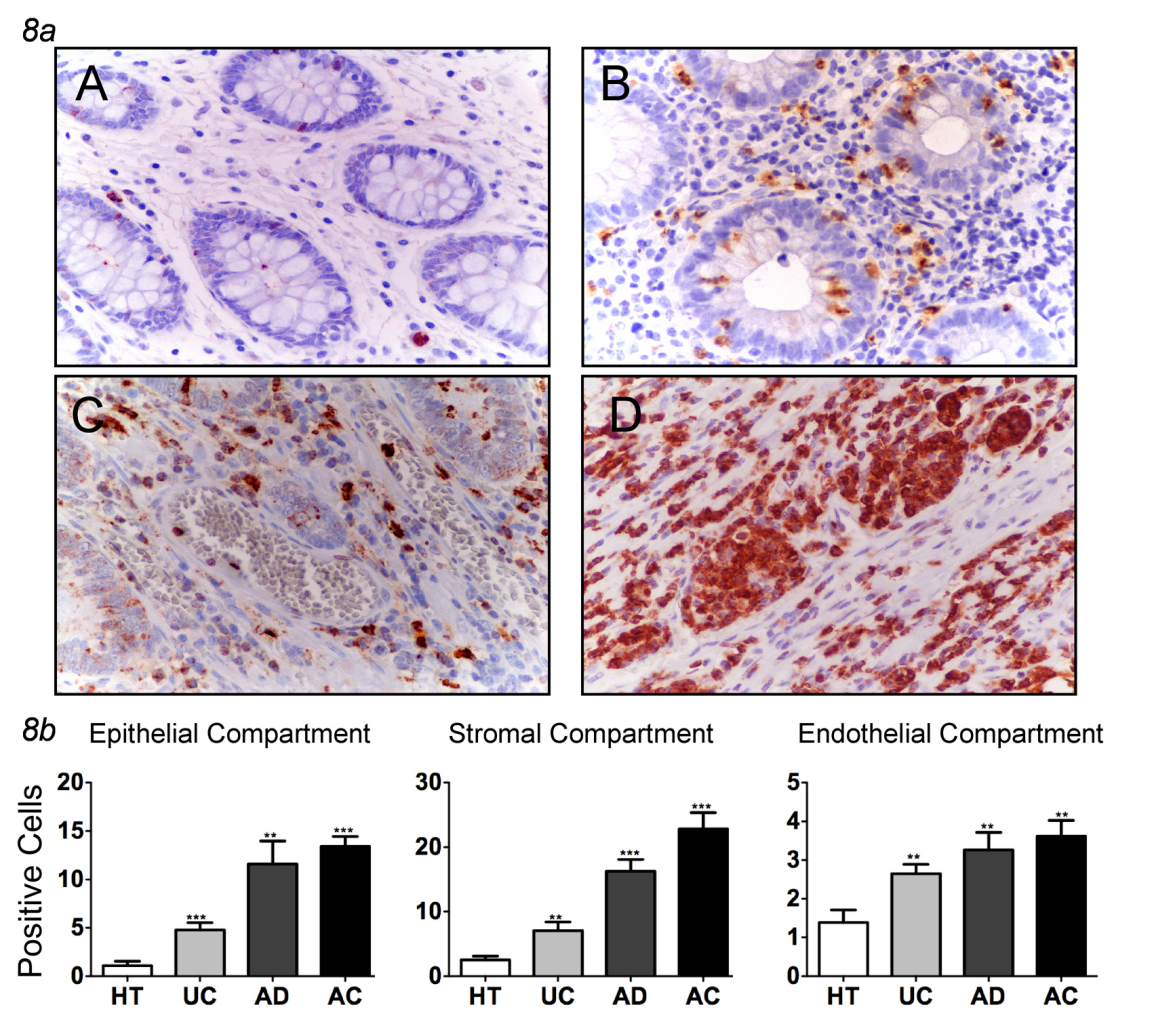
**CD15 expression in human colon tissues**. ***8a ***CD15 immunostaining in healthy tissues (A), ulcerative colitis (B), adenomas (C) and adenocarcinomas (D). The figure shows an increasing expression in all the compartments: endothelial, epithelial and stromal. In the AC tissues there is a wide distribution and a strong intensity of the marker (magnification ×400). ***8b ***Expression of CD15 in three different compartment shows that in all groups CD15 is significantly increased respect to healthy tissues (mean ± SEM; **P < 0.01, *** P < 0.001). HT = healthy tissues (N = 16); UC = ulcerative colitis (N = 13), AD = adenomas (N = 34; 29 low and 5 high grade), AC = adenocarcinomas (N = 53; 7 T1, 10 T2, 33 T3, 3 T4).

This trend was even more evident when analyzing the distribution of CD15+ (Figure 8, Table 2). In this case clear compartment-specific differences were observed; the percentage of CD15+ cells present in the stromal and epithelial compartments of UC were significantly less than the number of CD15+ cells observed in the same compartments of AD, and AC tissues, respectively (p < 0.05 in all comparisons) (Figure 8, Table 2). In the healthy, UC and AD tissues, the staining pattern was largely associated with neutrophils, while CD15 expression was more widely distributed in the AC tissues.

### Angiogenesis markers with increasing tissue dysplasia

**Figure 9:**
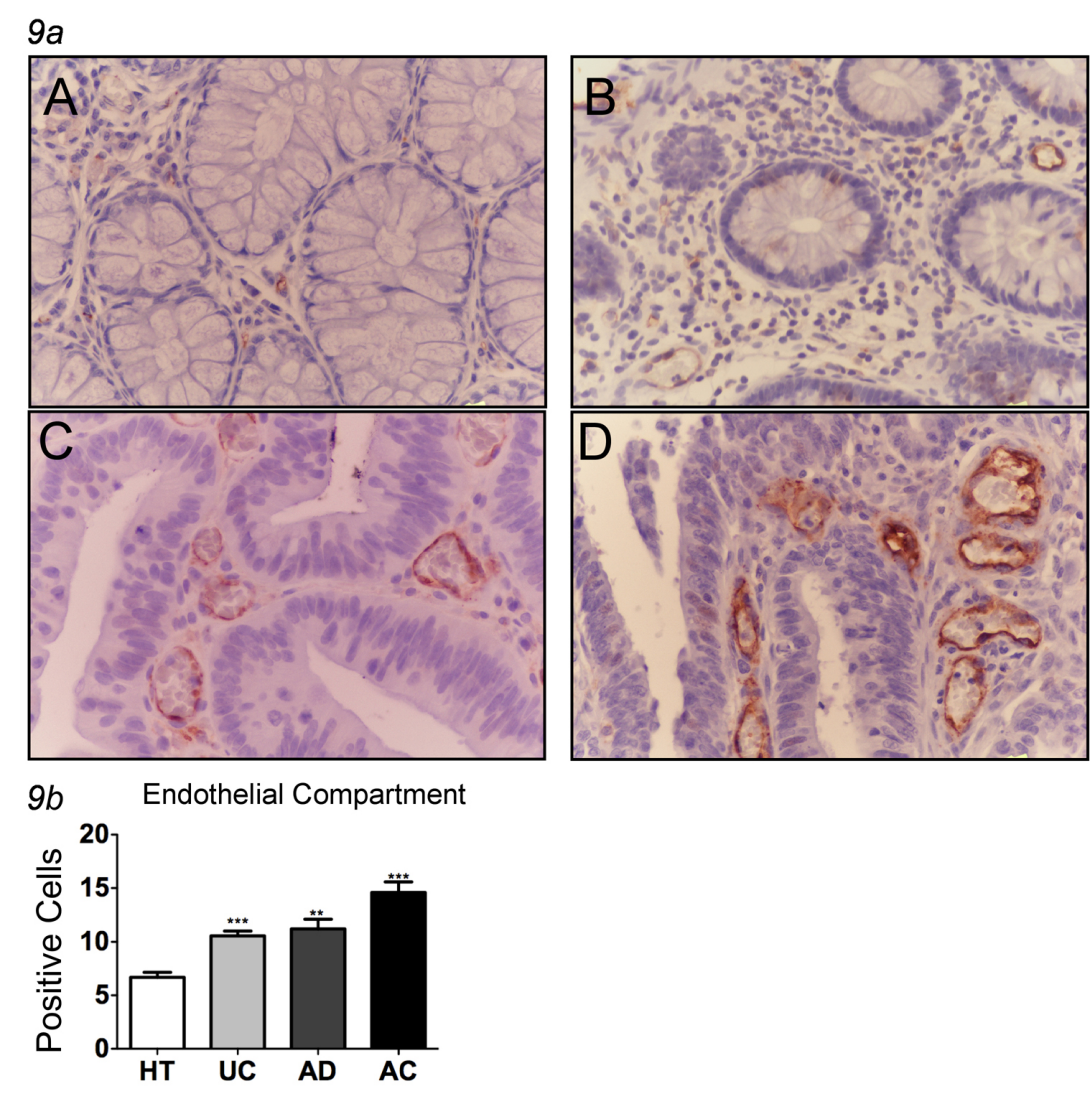
**CD31 staining in human colon tissues**. ***9a ***Immunohistochemistry for CD-31 in healthy tissue (A), ulcerative colitis (B), adenomas (C) and adenocarcinomas (D) showed a correlation between dysplastic condition and expression of the marker. From ulcerative colitis to adenocarcinoma there is an increase in vascular density and intensity of marker expression (magnification ×400). ***9b ***Different expression of CD-31 (in endothelial compartment) in healthy tissues, ulcerative colitis (UC), adenomas (AD) and adenocarcinomas (AC) show that the increasing grade of dysplasia directly correlates increased vascularization. In all groups CD31 is significantly increased with respect to healthy tissues (mean ± SEM; **P < 0.01, *** P < 0.001). HT = healthy tissues (N = 16); UC = ulcerative colitis (N = 13), AD = adenomas (N = 34; 29 low and 5 high grade), AC = adenocarcinomas (N = 53; 7 T1, 10 T2, 33 T3, 3 T4).

Immunostaining with anti-CD31 antibody showed an increased density of vessels, identified by the presence of a lumen, in pathological specimens compared with healthy tissues (Table 2). In particular, the percentage of CD31+ cells in healthy tissues was 6.67%, and it rose to 10.55% in UC (p < 0.05), 11.21% in AD (p < 0.05), and 14.59% in AC (Figure 9, Table 2). The observed percentage of CD31+ cells in adenocarcinoma specimens was twice that of controls (14.59%, P < 0.05), and it was significantly increased in comparison with the percentage of CD31+ cells observed in UC tissues (Figure 9, Table 2).

## Discussion

The tumor microenvironment is a complex network of different cell types and numerous intracellular mediators, including inflammatory and other immune cells, stromal, endothelial, and epithelial cells. These elements appear to actively participate in tumor progression and dissemination, where the tumor microenvironment not only responds to and supports carcinogenesis, but also contributes to tumor initiation, progression, and metastasis. The mutual interaction between transformed cells and the microenvironment modifies tumor fate.

Although neoplastic transformation in inflammatory bowel disease (IBD) is thought to be similar to the adenoma-carcinoma sequence in sporadic CRC, several differences exist. While in colitic mucosa the dysplasia is usually multifocal, suggesting a "field effect", in sporadic CRC the preneoplastic lesions are usually focal and mass forming. There are also several differences in the sequences of molecular events leading from dysplasia to invasion in adenocarcinoma arising in IBD as compared with sporadic CRC. For example, loss of APC function is a common and early event in sporadic CRC, while it is a much less frequent, and usually late, event in the colitis-associated dysplasia-carcinoma sequence. Further, in patients with colitis-associated cancer, p53 mutation is an early event that may also be detected in the non dysplastic mucosa, while it is late in sporadic CRC [52].

There is a clear relationship between chronic inflammation and colon cancer, however, the exact mediators by which chronic inflammation promotes colorectal carcinogenesis are still unclear. Persistent inflammation is believed to result in increased cell proliferation as well oxidative stress that leads to the development of dysplasia [9]. Oxidative stress is particularly intense in inflammatory conditions, largely due to extensive neutrophil and macrophage recruitment. These cells become activated in the inflamed tissue and produce substantial quantities of reactive oxygen species (ROS) and reactive nitrogen (RON), leading to DNA damage, including gene mutations, genetic instability and aberrant methylation. RONs may interact with genes involved in colorectal carcinogenetic pathways such as p53, DNA mismatch repair genes and other factors such as NF-κB and COX-2 [21,53-55].

Here we studied the expression patterns of selected inflammatory and angiogenesis markers in tissue specimens with increasing tissue dysplasia into colorectal tumor progression. Analyzing samples from predisposing conditions (such as ulcerative colitis) to neoplastic pre-cancerous lesions to invasive cancer, we detected a significant increase in angiogenesis using CD31 staining, inflammatory cells expressing CD68 or CD15, cytokines like IL-6 and other mediators that play a key role in the innate immune system, in particular TLR4. Further, a distinctive pattern of cells and cytokines within the tissue compartments, tumor and microenvironment, could be identified. Specifically, we found a more intense staining for all the inflammatory markers in the stromal compartment of AC samples, indicating that these major players of inflammation infiltrate tumor tissues. High levels of tumor infiltration by T cells (using CD3) or memory T cells (CD45RO) in both the invasive margin and tumor center has been associated with better clinical outcome [25], suggesting that these could be useful markers of prognosis. However, additional studies examining the postsurgical development of metachronous metastases indicated that levels of CD3+ cells infiltrating into the invasive margin was not an independent predictor of clinical outcome in patients with stage III colorectal cancer [56]. IL-6 activates a feed-forward loop leading to increased STAT3 activation in cancer and inflammatory cells [32], where STAT3 promotes polarization of innate immunity towards immuno-suppressive alternate activation. Our results indicate the innate response related to activation of the TLR4-IL6 axis found here would be associated with repression of adaptive anti-tumor immune responses.

We hypothesize a scenario where the microenvironmental contribution to tumor progression also could be segmented in a multistep process, the first step being the transition from healthy mucosa to ulcerative colitis, and corresponding to a massive inflammatory-angiogenic reaction. In ulcerative colitis, inflammation and angiogenic markers showed significantly higher expression than in healthy tissues. HGF, a mediator with significant anti-inflammatory activity [57], particularly in the gastrointestinal tract [58] where it appears to suppress inflammation by acting on NFκB pathway and affecting downstream factors such as TLR4-IL6 [57], was expressed at high levels only in the controls. In contrast, pro-inflammatory markers (IL6, CD68, CD15, TLR4) expression is higher in adenocarcinoma tissues respect to the adenoma, the ulcerative colitis and the healthy tissues.

Lack of control of the host reaction to tumor growth is essential for tumor progression. In addition to repressed HGF, we validated the enhancement of TLR-4-bearing and IL-6-secreting cells in adenocarcinoma specimens of colitis-associated cancer developed in mice lacking the expression of Tir8, an inhibitory member of the interleukin-1 receptor family that acts as a negative regulator of NF-κB activation in response to TLRs and interleukin 1 receptor agonists [59]. This correlates with the protection from AOM induced colorectal cancer in TLR4-/-mice [41], suggesting that TLR4 signaling plays a role in CRC progression.

Chronic inflammation and related abnormalities in the gut flora as observed in IBD, are associated with a higher incidence of colon cancer. TLR-4 is a key pattern recognition receptor that mediates innate immune responses to pathogen-associated molecules, most notably the lipopolysaccharides (LPS) of Gram-negative bacteria, triggering phagocyte activation and shaping adaptive immune responses [60]. TLR-4 also recognizes endogenous ligands produced by tissue damage, including fragments of extracellular matrix molecules such as hyaluronic acid, heparan sulfate, and proteoglycans, as well as intracellular proteins, in particular the proinflammatory high-mobility group box 1 (HMGB1) protein [60]. These ligands trigger inflammation and tissue repair responses [60], Akt activation [50] that is often associated with tumors and tumor progression [8,17,19]. Changes induced by unbalanced inflammation and bacteria could contribute to colon cancer development through release of LPS that binds TLR-4 present on the surface of inflammatory cells, and induce an inflammatory reaction. Consistent with an increased expression with advancing disease, TLR-4 expression was associated with different survival of patients with invasive colon AC. We observed that AC patients who had a percentage of TLR-4+ cells in the tumor stromal compartment, (mostly immune cells), lower than the median value had fewer relapses, and the relapses that occurred did so after a longer lag time that those AC patients with higher TLR-4 expression. Recent independent studies also found a correlation between TLR4 expression and patient survival in adenocarcinomas [61], although the study was limited to tumor cell expression and the role for these markers in less malignant conditions or other markers of inflammation were not investigated.

## Conclusion

Here we show enhanced expression of TLR-4 on cells of the epithelial and stromal tissue compartment as well as players in the inflammatory and angiogenic pathways are strongly increased during colorectal cancer progression. Our data corroborate the concept that inflammation correlates with the degree of malignancy in colon cancer and provides innovative data on the role of signaling by TLR-4 both in the tumor and the microenvironment. Accordingly, TLR-4 appears to have the potential to become a marker of disease progression in patients with colon malignancies or pre-malignant lesions.

## List Of Abbrevations

IL6: (Interleukin 6); TLR4: (Toll like receptor 4); HGF: (Hepatocyte Growth Factor); HT: (Healthy tissue); CRC: (Colon Rectal Cancer); UC: (Ulcerative Colitis); AD: (Adenoma); AC: (Adenocarcinoma).

## Competing financial interests statement

The authors declare that they have no competing interests.

## Authors' contributions

RC carried out experiments and wrote the manuscript. VB carried out the design of the study and performed statistical analysis. GP participated in writing the manuscript. EOB and OG participated in the research of tissue samples and clinical records. CG provided the murine experiments. LL helped to draft the manuscript. MCB and FS participated in the study design and in tissue analysis. DMN and AA wrote the manuscript, participated in study design and performed statistical analysis.

All authors have read and approved the final manuscript.

## Supplementary Material

Additional file 1 **Supplementary Table 1: Clinico-pathological information of patients**. listed are patients' ID number, clinical diagnosis, TNM stage, sex, age, and disease free survival in months. Click here for file
